# Shifts in Rhizosphere Bacterial Community Composition and Predicted Functional Potential Associated with *Impatiens parviflora* Invasion in Temperate Forest

**DOI:** 10.1007/s00248-026-02807-1

**Published:** 2026-06-12

**Authors:** Patryk Wiśniewski, Mateusz Maździarz, Kamila Kwietniewska, Katarzyna Krawczyk

**Affiliations:** 1 Department of Botany and Evolutionary Ecology, University of Warmia and Mazury in Olsztyn, 10‑719 Olsztyn, Poland; 2 Department of Immunology and Infectious Biology, Institute of Microbiology, Biotechnology and Immunology, Faculty of Biology and Environmental Protection, University of Lodz, 90-237 Lodz, Poland

**Keywords:** Bacterial diversity, Biological invasion, Microbiome, Small balsam, Soil ecology

## Abstract

*Impatiens parviflora* is a widespread invasive plant in temperate European forests, yet its influence on rhizosphere microbial communities remains poorly understood. This study provides initial metagenomic insights into taxonomic shifts and predicted functional potential of bacterial communities associated with this invader. Rhizosphere soils were collected from eight *I. parviflora*-invaded and eight non-invaded control plots in a mixed coniferous forest in northern Poland and analysed using Oxford Nanopore shotgun sequencing, with functional inference performed using the taxonomy-dependent FAPROTAX database. Bacterial richness was significantly higher in invaded soils, whereas Shannon and Simpson diversity indices did not differ between treatments, indicating an expansion of rare taxa without changes in overall diversity structure. The invaded rhizosphere was characterised by a uniform depletion of dominant bacterial orders, with no significantly enriched taxa detected, contrasting with the selective enrichment of microbial groups often reported for other invasive plant species. FAPROTAX-based predictions indicated consistently lower inferred abundances of 37 metabolic processes in invaded plots, including those related to nitrogen cycling and degradation of complex plant polymers. Because these functional predictions are derived from taxonomic composition, they represent inferred ecological potential rather than measured activity. Overall, these results generate testable hypotheses regarding plant–soil feedbacks and highlight the utility of long-read metagenomics for exploring microbial dynamics potentially contributing to the ecological success of *I. parviflora* in temperate forests.

**Supplementary Information:**

The online version contains supplementary material available at 10.1007/s00248-026-02807-1.

## Introduction

Globalization, climate change, and other human-driven factors increasingly facilitate the spread of alien species, making biological invasions a major cause of biodiversity loss and ecosystem disruption [1, 2]. Many introduced plants become naturalized or invasive, often owing their ecological success to traits such as rapid growth, high reproductive output, and allelopathic potential, which confer significant competitive advantages over native flora [1, 3]. Among the invasive plants spreading across temperate Europe, *Impatiens parviflora* DC. (small balsam) is particularly noteworthy for its broad ecological tolerance and high reproductive capacity.

*I. parviflora* is an annual plant native to Central Asia that has become highly adaptable in temperate Europe, where it now occurs widely across Central Europe, Scandinavia, and the Baltic states. The species thrives in diverse habitats, including coniferous and mixed forests as well as open non-forest areas, particularly in sites with moderate to high nutrient availability. In such environments, it can achieve rapid growth and strong competitive performance [4]. In temperate forests, *I. parviflora* thrives in nitrogen-enriched, disturbed sites where ruderalization promotes the expansion of nitrophilous species, notably the competitive clonal plant *Aegopodium podagraria* [5, 6]. Its ecological success is further supported by a high reproductive capacity, as individual plants produce between 2,000 and 10,000 seeds that can remain viable in the soil for up to five years [7]. Notably, *I. parviflora* exhibits the strongest allelopathic activity among European *Impatiens* species – leaf extracts rich in phenolic compounds inhibit seed germination and root growth of neighbouring plants, often surpassing even that of the aggressive native competitor *Urtica dioica* [8, 9]. Importantly, *I. parviflora* has been shown to suppress native closely related species such as *Impatiens noli-tangere*, particularly under resource-limited conditions [10]. It may also indirectly affect economically important plants; [11] reported that interactions between alien balsams, strawberries (*Fragaria × ananassa*), and pollinators can influence strawberry reproductive success. A further peculiarity of *I. parviflora* is its counter‑intuitive nitrogen response: unlike the native *I. noli-tangere*, plant height and seed production per capsule in *I. parviflora* decrease with increasing soil nitrogen availability, pointing to a distinct nutrient‑use strategy [12].

Invasive plants impact soil ecosystems not only by directly modifying habitats but also by shaping the composition and functioning of soil microbial communities [13, 14]. Soil microbiota are dynamic and functionally diverse, strongly influencing plant establishment, growth, and competitive interactions [15]. The rhizosphere is particularly important in this context, as it represents a highly dynamic interface where plant-driven selection processes directly shape microbial assemblages through root exudation and plant–soil feedbacks [16, 17]. In contrast to the phyllosphere and endosphere, which are more strongly influenced by atmospheric conditions and internal plant tissues, respectively, the rhizosphere is continuously modulated by root exudation and soil feedbacks. It therefore represents the primary zone where plant invasion effects on microbial communities are expected to be most pronounced [16, 18].

Through root exudates and litter inputs, invasive plants may influence microbial community structure. Such microbially mediated plant–soil feedbacks are increasingly recognized as key drivers of invasion success [19–21]. Plant-associated microbiomes are organized into distinct communities inhabiting the rhizosphere, phyllosphere, and endosphere. In several invasive plant systems, microbial partners have been shown to enhance growth, adaptability, and resistance to biotic stress [22, 23]. For instance, members of Actinobacteria and *Burkholderia* spp. contribute to nutrient mobilization, pathogen suppression, and stabilization of plant–soil feedbacks [24–26]. Similar mechanisms have been observed in other invasive plant systems, such as *Solidago canadensis*, where shifts in rhizosphere microbial communities, particularly involving arbuscular mycorrhizal fungi (AMF), enhance plant growth and competitive ability [27, 28]. Likewise, *Fallopia japonica* has been shown to alter soil biota and belowground interactions, potentially influencing ecosystem processes [29]. For *I. parviflora* itself, arbuscular mycorrhizal (AM) symbiosis has been directly shown to improve plant vigour, flowering, and fruit production; mycorrhizal colonization correlates positively with soil pH and negatively with the soil C/N ratio, demonstrating that AM fungi actively contribute to the species’ invasive success in temperate forests [30]. Moreover, the invader restructures the soil nematode community, increasing the relative abundance of bacterivores while reducing plant‑parasitic nematodes, which points to a maturing, bacteria‑dominated decomposition pathway in the soil food web [14].

Given the ecological importance of plant–microbe interactions, it is plausible that *I. parviflora* is associated with specific microbial assemblages that may contribute to its competitive performance in invaded ecosystems. However, plant-associated microbiomes often differ between native and invaded ranges, and comparisons between invaded and non-invaded systems are inherently confounded by host plant identity [31, 32]. Because *I. parviflora* is, by definition, absent from non-invaded plots, control samples must be collected from the rhizosphere of native vegetation. Consequently, observed differences in microbial communities may reflect both invasion status and plant-specific effects rather than invasion alone [33]. To address this, we employed a paired sampling design that minimized environmental variation, while also characterizing habitat conditions using Ellenberg indicator values and soil pH.

Therefore, characterizing microbiomes associated with *I. parviflora* can provide important insights into invasion mechanisms, ecosystem-level feedbacks, and potential management strategies [2]. Despite growing interest in plant–microbe interactions, studies explicitly addressing the rhizosphere microbiome of *I. parviflora* remain extremely limited, and no studies to date have applied long-read metagenomic approaches to investigate this system in temperate European forests.

Advances in high-throughput, genome-resolved approaches have become increasingly valuable in invasion ecology, as identifying microbial taxa linked to plant dominance requires cultivation-independent methods [34]. Whole-metagenome shotgun (WMS) sequencing enables simultaneous characterization of the taxonomic structure of complex environmental microbiomes [35]. Functional profiling based on taxonomic composition is a widely used approach in microbial ecology, particularly in soil studies. Tools such as FAPROTAX assign putative ecological functions to prokaryotic taxa based on manually curated, literature-derived associations rather than direct gene-level evidence, and thus provide a proxy for functional interpretation of community composition in environmental microbiomes [36, 37].

In this context, Oxford Nanopore long-read sequencing improves the reconstruction of soil microbial communities by enabling more accurate recovery of full-length taxonomic markers relevant to plant–soil interactions, thereby enhancing the resolution of microbial diversity and supporting ecological inference. At the same time, Nanopore sequencing is associated with higher error rates compared to short-read technologies, which may influence taxonomic classification accuracy and downstream analyses. However, these limitations are continuously being reduced through methodological improvements [38, 39].

In our research we aimed to: (i) characterize the taxonomic structure of rhizosphere-associated microbial communities; (ii) identify microbial taxa potentially associated (based on taxonomic inference) with nutrient cycling and plant–microbe interactions; and (iii) assess microbial taxa that may be associated with the ecological success of *I. parviflora*. Our results provide new insights into the role of soil microbiomes in supporting invasion strategies of *I. parviflora* and their potential impacts on European ecosystems.

## Materials and Methods

### Study Area and Site Characteristics

The study was conducted in northern Poland (Warmian–Masurian Voivodeship, Olsztyn County), within the Kudypy Forest District (Redykajny Forest Range), in a compact forest area near Lake Długie (Olsztyn), located in the Olsztyn Lakeland (Masurian Lake District) according to the physico-geographical regionalization of Poland [40]. Sampling plots were distributed across multiple forest patches within this area, within the geographic extent of approximately 53.786–53.793° N and 20.452–20.466° E, with the centroid of the study area located at approximately 53.7905° N, 20.4589° E. The elevation of the sampling sites ranged from 100 to 150 m a.s.l.

The regional climate is temperate (Köppen–Geiger classification: Dfb [41]), with a transitional character between oceanic and continental conditions. Olsztyn has a temperate transitional climate with a distinct continental influence. The mean annual air temperature is approximately 8.2 °C. The warmest month is July, with a mean temperature of 18.8 °C (average maxima around 22.7 °C), while January is the coldest, with a mean of − 2.8 °C and minima down to − 5.0 °C. Annual precipitation totals about 715 mm, with a summer maximum (up to 92 mm in July) and a winter minimum (around 44–52 mm), indicating a clear seasonal pattern in both temperature and rainfall [42].

The study was conducted in a fresh mixed coniferous forest (Polish classification: bór mieszany świeży, BMśw). The dominant tree species included *Pinus sylvestris*, *Fagus sylvatica*, *Picea abies*, and *Corylus avellana*. The composition of the herb layer was recorded in all plots.

Soil classification was assessed using publicly available spatial soil databases (WRB). According to SoilGrids data, the study area is predominantly covered by Cambisols, with a local occurrence of Podzols. Cambisols represent weakly to moderately developed soils, typically forming under temperate climatic conditions on a variety of parent materials. In contrast, Podzols occur locally in areas affected by stronger podzolization processes, usually associated with acidic parent material and coniferous or mixed forest cover. Field measurements (pH 5.0–5.5) are consistent with generally acidic soil conditions observed in parts of the study area.

*Impatiens parviflora* is a widespread and well-established invasive species in Central Europe and has been present in the study region for decades. Field observations confirm its occurrence at the sampling sites since at least 2018 (unpublished data).

**Fig. 1:**
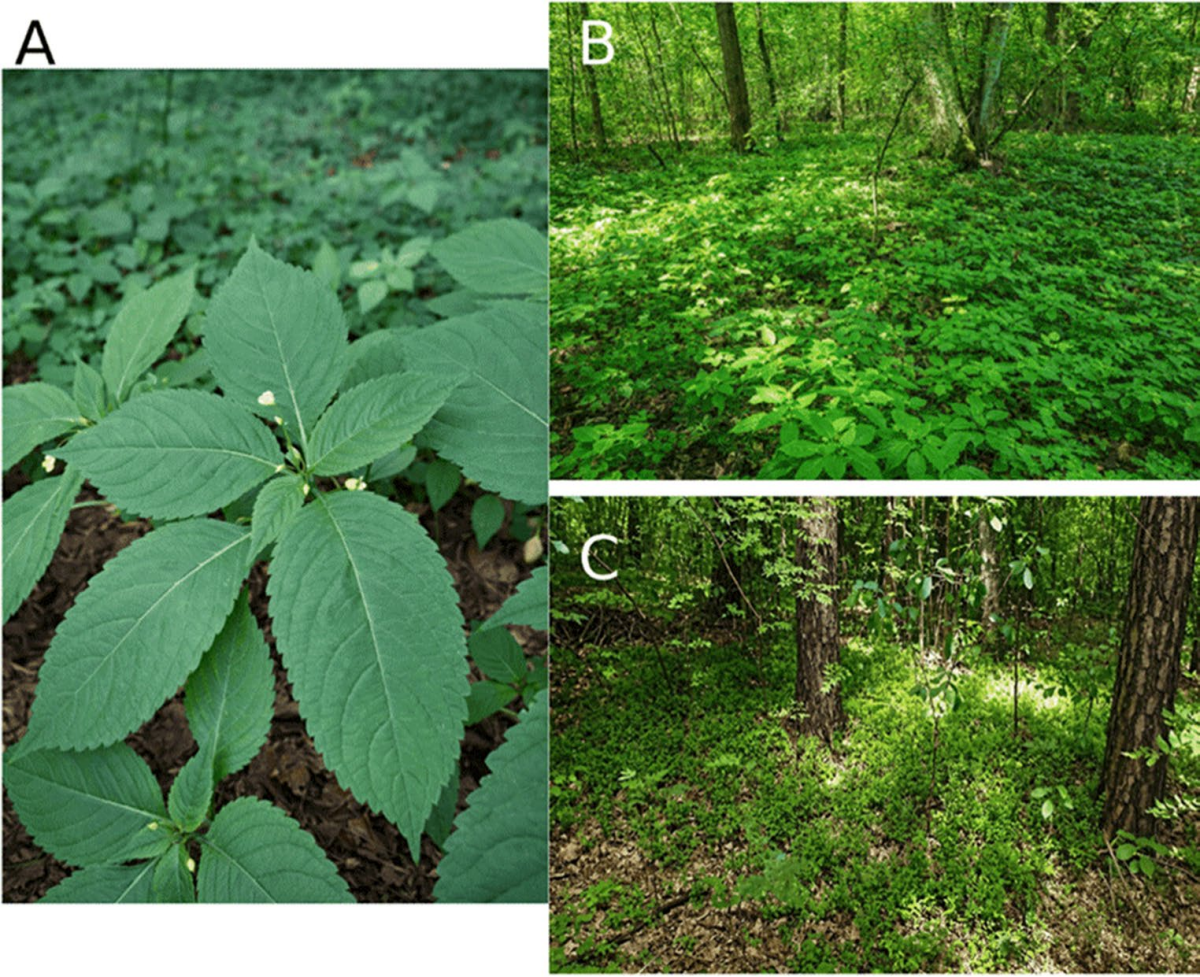
(**A**) *Impatiens parviflora* DC., characteristic serrated leaves and developing flowers visible. (**B**) *Impatiens parviflora* DC., habit of the plant in a forest understory. (**C**) control surface (without invasion of *Impatiens parviflora* DC.). Photograph by Patryk Wiśniewski

Characteristic serrated leaves and developing flowers of *I. parviflora* are shown in Fig. 1A, while its growth habit in the forest understory is illustrated in Fig. 1B; the control surface without the presence of this species is presented in Fig. 1C.

### Field Sampling and Soil Collection

Field sampling was carried out in August 2022 under stable mid-summer conditions. A total of 16 sampling plots (10 × 10 m) were established using a paired design, consisting of 8 invaded plots with high cover of *Impatiens parviflora* (> 75%, corresponding to Braun-Blanquet [43] class 5) and 8 non-invaded control plots where the species was absent. Each invaded plot was paired with a nearby control plot located within a similar microhabitat (light availability, slope, and vegetation structure) in order to minimize environmental variability, with a minimum distance of 30 m between plots to ensure spatial independence.

In each plot, vegetation surveys were conducted using the Braun-Blanquet method [43], during which species composition and cover-abundance values were recorded for all vascular plants in the herb layer.

Rhizosphere soil samples were collected in both invaded and control plots from the 0–10 cm mineral soil layer using sterile tools (shovel and brushes). In invaded plots, samples were taken from the rhizosphere of *Impatiens parviflora*, while in control plots they were collected from the rhizosphere of the native understory species. At each plot, rhizosphere soil was collected from several individual plants by carefully sampling soil tightly adhering to the roots after gentle removal of bulk soil. The subsamples were pooled to form one composite sample per plot.

All tools were disinfected with ethanol between samples, and disposable gloves were changed between plots to prevent cross-contamination. Each composite sample was used to fill a 5 mL Eppendorf tube. Immediately after collection, samples were stored in a portable cooler at 4 °C, transported to the laboratory, sieved through a 2 mm mesh to remove debris, and processed on the same day, with DNA extraction performed directly after homogenization.

### Soil pH and Ellenberg Indicator Values

Soil pH was the only physicochemical parameter directly measured and ranged from 5.0 to 5.5 across all plots, indicating relatively homogeneous acidity conditions. Due to logistical constraints, additional soil chemical analyses (e.g., organic carbon, nitrogen, phosphorus, soil organic matter, or texture) were not performed.

Ecological conditions of the study plots were assessed using Ellenberg indicator values [44], based on vascular plant species composition. Indicator values for light (L), soil moisture (F), and nutrient availability (N) were assigned to each species according to standard Central European Ellenberg ecological indicator tables.

Species cover-abundance was recorded using the Braun–Blanquet scale and subsequently converted into semi-quantitative numerical values (*r* = 0.1, + = 0.5, 1 = 1, 2 = 3, 3 = 5, 4 = 7.5, 5 = 9) to allow quantitative analysis. For each plot, weighted mean Ellenberg indicator values were calculated using species cover as weights.

To minimize the effect of strong dominance by the invasive species *Impatiens parviflora*, analyses for invaded plots were performed both including and excluding this species. For comparative analyses, values excluding *I. parviflora* were used. Plots 1–8 were treated as control sites, while plots 9–16 represented invaded sites. Differences in Ellenberg indicator values (L, F, N) between control and invaded plots were tested using t-tests for independent samples. Normality and homogeneity of variances were verified using the Shapiro–Wilk and F-tests, respectively.

### DNA Extraction and Quantification

Genomic DNA was extracted from all 16 soil samples using the PureLink™ Microbiome DNA Purification Kit (Invitrogen) according to the manufacturer’s instructions. The protocol was modified by reducing the volume of elution buffer S6 from the recommended 100 µl to 50 µl and by increasing the incubation temperature from room temperature to 37 °C in order to improve DNA yield.

DNA concentration and purity were initially assessed spectrophotometrically using an Agilent Cary 60 UV–Vis spectrophotometer. Fluorometric quantification was subsequently performed using a Qubit 4 Fluorometer (Invitrogen) following the manufacturer’s guidelines.

To further improve DNA quality, extracts were purified using the Monarch^®^ Genomic DNA Purification Kit (New England Biolabs). After purification, both spectrophotometric and fluorometric measurements were repeated.

### Library Preparation and Whole-Genome Nanopore Sequencing

Whole-genome shotgun (WGS) sequencing libraries were prepared using the Ligation Sequencing gDNA – Native Barcoding Kit 24 V12 (SQK-NBD114.24; Oxford Nanopore Technologies, UK) following the manufacturer’s protocol. DNA samples were barcoded, end-repaired, ligated, and purified using magnetic bead-based cleanup steps recommended for high-molecular-weight DNA.

Sequencing was performed on an Oxford Nanopore MinION Mk1C device equipped with R10.4.1 flow cells. Barcoded WGS libraries were loaded onto the flow cell according to Oxford Nanopore Technologies guidelines and sequenced until pore depletion.

In the subsequent stage, bioinformatic analyses were performed. Raw sequencing data generated by the MinION Mk1C sequencer and stored in FAST5 format were basecalled and converted to FASTQ files using the Guppy basecalling algorithm integrated into the MinKNOW software (v. 3.6.1). Basecalling was performed using the “super-accurate” model, which provides the highest read accuracy.

### Identification of Organisms from Nanopore Reads

High-quality reads were obtained using the chopper program v.0.12.0b [45] with a -q 7 and -l 500 flags. Subsequently, sequencing statistics were verified using FastQC v.0.11.9 [46] (https://www.bioinformatics.babraham.ac.uk/projects/fastqc/*)* and summarized with MultiQC v.1.14 [47]. Identification of organisms within the control samples (F_C) and *I. parviflora* rhizosphere samples (F_Ip) was conducted using the wf-metagenomics Nextflow pipeline (https://github.com/epi2me-labs/wf-metagenomics*)* with Standard-8 database, which provides a high-accuracy metagenomic classification for nanopore sequencing reads. To facilitate downstream analysis, previously generated Kraken reports were converted into the BIOM table format using kraken-biom v1.0.1 (https://github.com/smdabdoub/kraken-biom*).* The BIOM format was analyzed using MicrobiotaProcess v.1.22.1 [48]. To properly account for the inherently compositional nature of the metagenomic dataset and avoid the biases associated with rarefying raw count data [49, 50], a compositionally aware data analysis (CoDA) framework was applied for beta-diversity evaluation. Zero counts were handled by adding a pseudocount (+ 0.01) to the count matrix, and the data were subsequently transformed using the Centered Log-Ratio (CLR) transformation. Beta-diversity structure was explored using Principal Component Analysis (PCA) based on the Aitchison distance (calculated as the Euclidean distance on CLR-transformed abundances). Analyses of differential abundance focused on the order level, as this represents a practical compromise between taxonomic resolution and classification confidence with long-read Nanopore data, while still capturing ecologically coherent groups [51]. The resulting abundance data were analyzed with wilcoxon test from stats package v.4.5.2 with bonferroni correction, where a significance threshold of adjusted p-value (p.adj) < 0.05 and absolute value of log2FoldChange (Log2FC) > 1 were established to identify taxonomic differences at the order level between F_C and F_Ip groups. Taxa with a log2FC > 1 were considered upregulated (more abundant in the F_Ip group), whereas those with a log2FC < −1 were classified as downregulated.

### Functional Annotation

Functional annotation, including metabolic and other ecologically relevant roles, was performed using the FAPROTAX database [52]. This analysis was carried out using the previously generated BIOM file and the collapse_table.py script with the following parameters: --output_format_collapsed classical, -n ‘columns_before_collapsing_excluding_unassigned’, and --collapse_by_metadata ‘taxonomy’. Subsequently, the identified processes were subjected to the Wilcoxon rank-sum test to evaluate differences between the F_C and F_Ip samples. P-values were adjusted using the False Discovery Rate (FDR) method, and processes with an FDR < 0.05 were considered statistically significant.

### Microbial Diversity Analysis

Alpha diversity was assessed using richness (observed number of taxa), the Shannon diversity index, and the Simpson diversity index (1–D). Two groups were compared: control samples (F_C) and *I. parviflora* rhizosphere samples (F_Ip), each with eight biological replicates (*n* = 8). Differences between groups were tested using the Wilcoxon rank-sum test, chosen because of the small sample size and non-normal distribution of the data. Statistical significance was set at *p* < 0.05.

### Visualizations

All visualizations were prepared using ggplot2 v.4.0.2 [53], ComplexHeatmap v.2.26.1 [54] and circlize v.0.4.17 [55].

## Results

### Ellenberg Indicator Values

Weighted Ellenberg indicator values differed between control plots (1–8) and invaded plots (9–16) (Table 1). Control plots were characterized by mean values of L = 5.71 ± 0.19, F = 5.49 ± 0.17, and *N* = 4.38 ± 0.17, whereas invaded plots (excluding *Impatiens parviflora*) showed values of L = 5.55 ± 0.10, F = 5.95 ± 0.10, and *N* = 5.55 ± 0.18.

**Table 1:** Mean Ellenberg indicator values for control plots and invaded plots, calculated without *Impatiens parviflora*

Indicator	1–8 (control)	9–16 (invaded, without Impatiens)	*p*-value
L	5.71 ± 0.19	5.55 ± 0.10	0.059
F	5.49 ± 0.17	5.95 ± 0.10	1.55 × 10⁻⁵
N	4.38 ± 0.17	5.55 ± 0.18	5.54 × 10⁻⁹

*L* light availability, *F* soil moisture, *N* nutrients

Invaded plots showed lower light availability (L) and higher soil moisture (F) and nutrient availability (N) than control plots; differences were significant for F and N (both *p* < 0.001), but not for L (*p* = 0.059). The floristic composition of the herb layer is presented in Supplementary Table S1.

### Bacterial Community Diversity and Structure

**Fig. 2:**
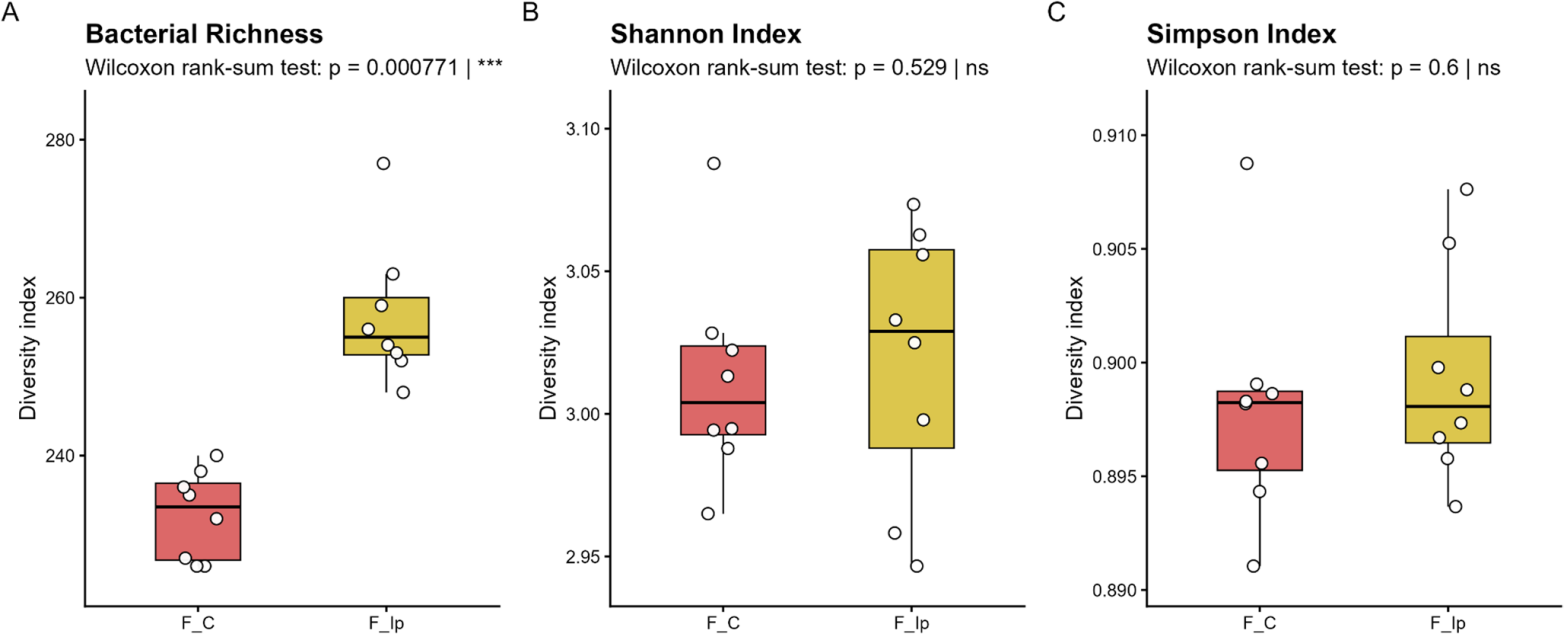
Impact of *Impatiens parviflora* invasion on soil bacterial alpha diversity. Comparison of biodiversity metrics between F_C and F_Ip groups. (**A**) Bacterial Richness shows a highly significant increase in the invasive group (Wilcoxon rank-sum test, *p* = 0.000771). Conversely, (**B**) Shannon Index (*p* = 0.529) and (**C**) Simpson Index (*p* = 0.6) reveal no statistically significant differences (ns) in diversity between the control and invaded sites. Box-plots represent the median, quartiles, and range, with individual data points plotted as open circles

Significant shifts in community composition associated with the invasion of *Impatiens* were partially revealed by the analysis of bacterial biodiversity. Regarding alpha diversity, the F_Ip exhibited a significantly higher Bacterial Richness compared to the F_C (257.8 ± 3.18 vs. 232.5 ± 1.98; *p* = 0.000771; Fig. 2A). Conversely, no statistically significant differences were observed between the control and invasive groups for the Shannon index (3.012 ± 0.0130 vs. 3.019 ± 0.0168; *p* = 0.529; Fig. 2B) and the Simpson index (0.8980 ± 0.00182 vs. 0.8994 ± 0.00169; *p* = 0.600; Fig. 2C)..

### Taxonomic Composition and Beta-Diversity of Rhizosphere Bacterial Communities

**Fig. 3:**
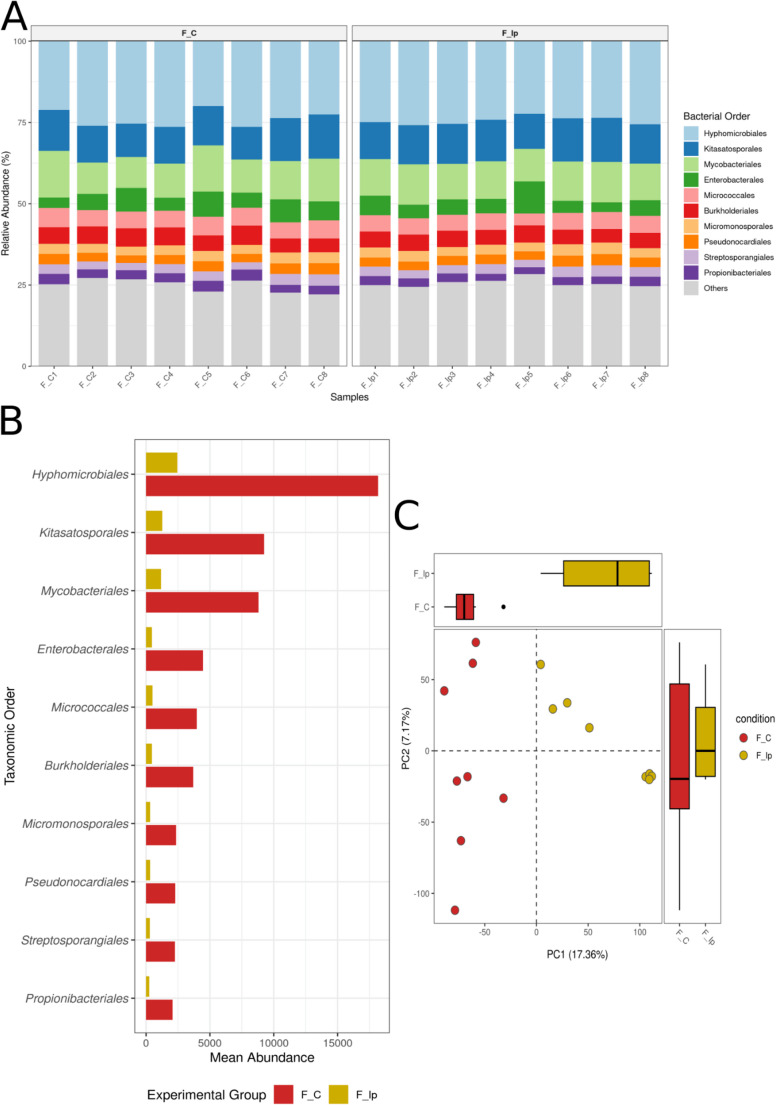
Taxonomic composition and multivariate statistical analysis of soil bacterial communities. (**A**) Relative abundance of bacterial orders across individual samples. The stacked barplot represents the percentage contribution of each bacterial order. The top 10 most abundant orders were highlighted using the color palette indicated in the legend, while all remaining orders were aggregated into the “Others” category (gray). (**B**) Mean abundance of dominant taxonomic orders. The barplot displays the mean abundance (x-axis) for selected taxonomic orders (y-axis). Groups were color-coded to distinguish between control samples (F_C, red) and *I. parviflora*-associated samples (F_Ip, yellow). (**C**) Principal component analysis (PCA) based on the Aitchison distance of bacterial profiles. The scatter plot illustrates the distribution of samples based on the first two principal components (PC1 and PC2). Samples were colored by group, with F_C represented in red and F_Ip in yellow

High-quality reads were yielded by the metagenomic sequencing of 16 soil samples (8 from control plots and 8 from the rhizosphere of *I. parviflora*), revealing diverse bacterial communities dominated by the phyla Proteobacteria, Actinobacteria, and Acidobacteria. Sequencing quality metrics indicated consistently high read quality across all samples after filtering (q > 7), with low levels of duplication (0.48–2.36%), stable GC content (60–63%), and variable sequencing depth among samples (Table S2). Sequencing depth differed substantially between libraries, reflecting variation in library yield rather than sequencing quality. Across the entire dataset, the mean sequencing depth was 153,689 reads per sample. At the order level, the ten most abundant bacterial taxa were Hyphomicrobiales, Kitasatosporales, Mycobacteriales, Enterobacterales, Micrococcales, Burkholderiales, Micromonosporales, Pseudonocardiales, Streptosporangiales, and Propionibacteriales (Fig. 3A, B). Differential abundance analysis indicated that all of these dominant orders were significantly less abundant in soils associated with *Impatiens parviflora* (F_Ip) than in the control soils (F_C) (Supplementary Table 3). All detected changes were negative, with log₂ fold changes ranging from − 2.87 to − 3.27 and p.adj of 0.0466. Among the most abundant orders, the strongest decreases were observed for Enterobacterales (log2FC = − 3.27), Propionibacteriales (log2FC = − 3.04), Burkholderiales (log2FC = − 2.99), and Micrococcales (log2FC = − 2.98). Similarly pronounced reductions were found for Hyphomicrobiales (log2FC = − 2.89), Pseudonocardiales (log2FC = − 2.90), Streptosporangiales (log2FC = − 2.90), Mycobacteriales (log2FC = − 2.91), Micromonosporales (log2FC = − 2.89), and Kitasatosporales (log2FC = − 2.87).

Principal Component Analysis (PCA) based on the Aitchison distance revealed a separation between F_C and F_Ip soil communities (Fig. 3C). The separation occurred primarily along the first principal component (PC1), which explained 17.36% of the total variance, with F_C samples clustering on the negative side of the axis and F_Ip samples on the positive side. The second principal component (PC2) accounted for an additional 7.17% of the variance. Control samples exhibited greater dispersion, particularly along PC2, whereas the F_Ip samples formed a more compact cluster.

### Differential Abundance of Bacterial Orders

**Fig. 4:**
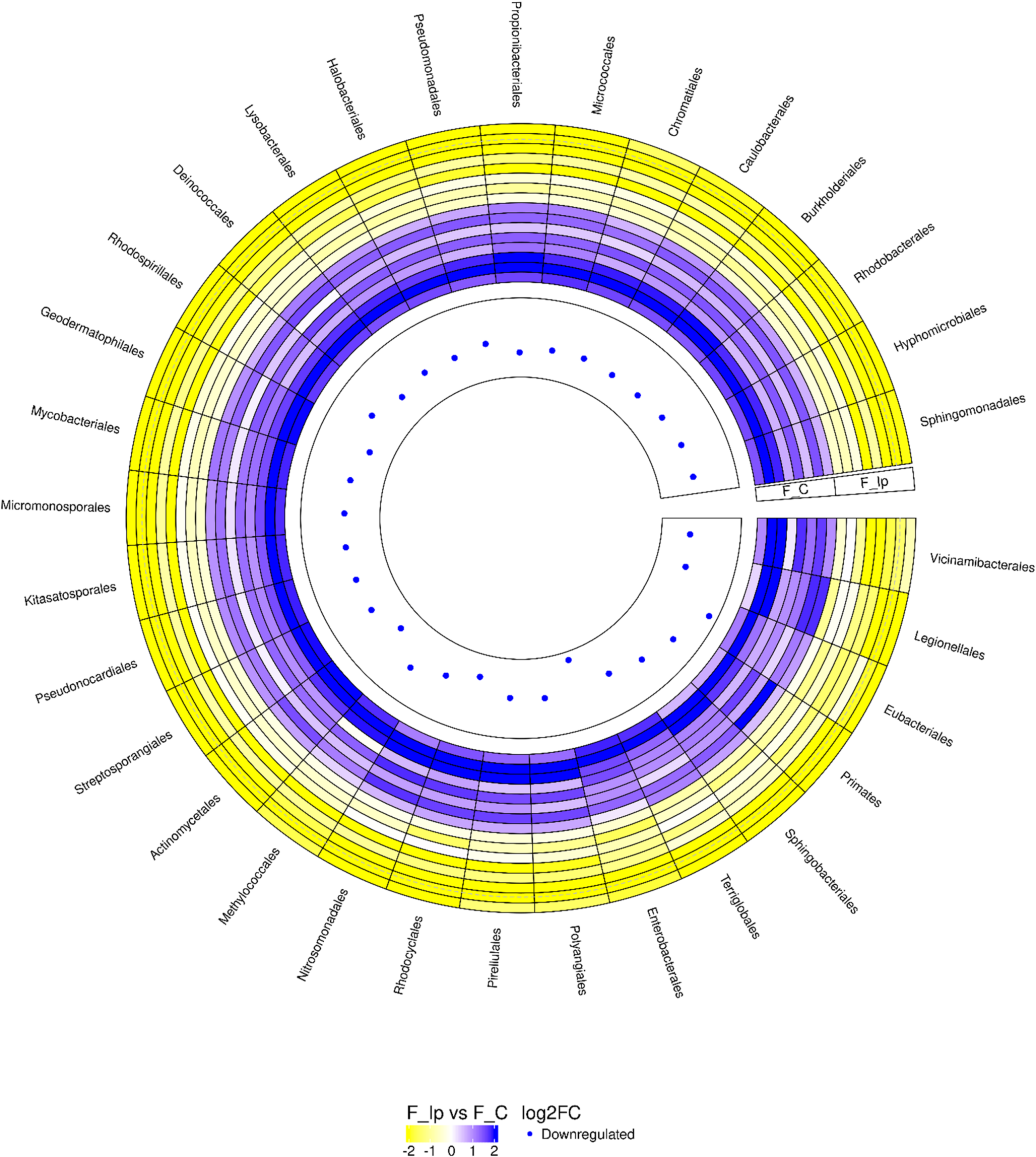
Circular heatmap representation of significantly differential bacterial orders between F_C and F_Ip samples. The heatmap displays bacterial taxa that exhibited statistically significant differences between the two groups (F_C vs. F_Ip). The color gradient represents the Z-score normalized abundance values, where darker blue shades indicate higher Z-scores (increased abundance) and yellow shades indicate lower Z-scores (decreased abundance). The specific color-to-value mapping is provided by the scale located in the center of the circular plot. The second track describes the log2FC of downregulated (blue) taxa

To identify specific bacterial orders driving community differentiation, differential abundance analysis was performed (Supplementary Table 3, Fig. 4). A total of 32 bacterial orders showed statistically significant differences in abundance between the control (F_C) and *I. parviflora* rhizosphere (F_Ip) samples (p.adj < 0.05). Notably, all statistically significant shifts were characterized by negative log2FC values, indicating that all detected differences reflected reduced abundance of these taxa in invaded soils. Among these significantly downregulated taxa, Burkholderiales, Propionibacteriales, Hyphomicrobiales, Kitasatosporales, and Pseudonocardiales exhibited some of the strongest responses, with log2FC ranging from − 2.89 to − 3.04 (Fig. 4).

### Widespread Suppression of Microbial Functional Potential in the Invaded Rhizosphere

**Fig. 5:**
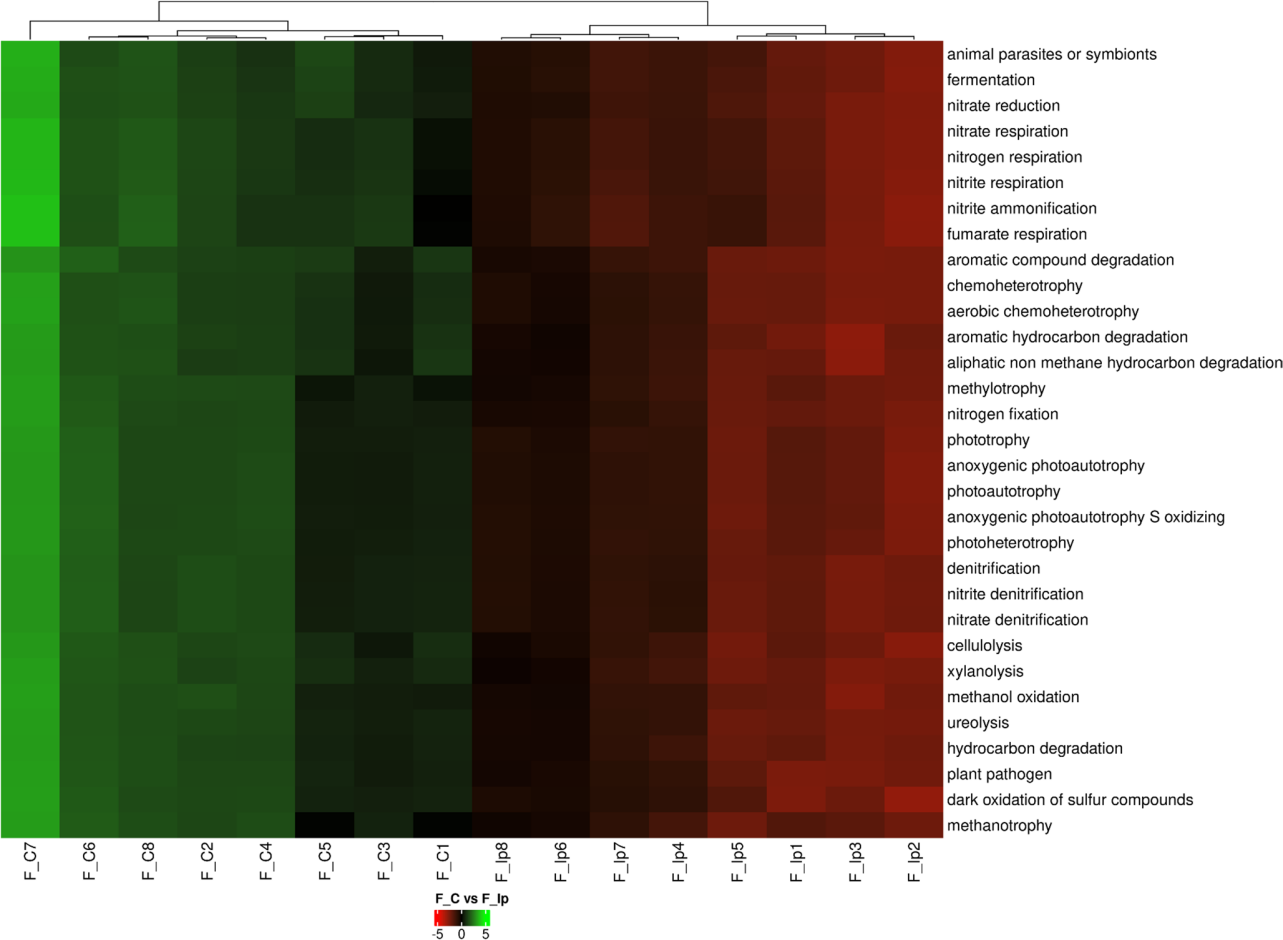
Heatmap of selected FAPROTAX functional processes differentiating F_C and F_Ip samples. The color gradient corresponds to Z-score normalized values, where intense green indicates a higher Z-score and intense red represents a lower Z-score. The color-to-value mapping is defined by the scale located at the bottom of the plot

The most notable pattern was a consistent reduction in the predicted functional potential of the microbial community associated with *I. parviflora*. Using the FAPROTAX database, 37 metabolic processes were identified with significantly different predicted abundances between the two groups (Fig. 5, Supplementary Table 4). The direction of change was uniform across all 37 processes: consistently higher Z-scores were recorded in the F_C compared to the F_Ip (Fig. 5). These results suggest a widespread reduction in the inferred metabolic capacity of the rhizosphere microbiome in invaded plots.

Key biogeochemical cycles were encompassed by this functional suppression. All processes associated with nitrogen cycling, including nitrogen fixation (log2FC = − 2.86), nitrate reduction (log2FC = − 3.11), denitrification (log2FC = − 2.91), and nitrate respiration (log2FC = − 3.23), were significantly depleted in the *I. parviflora* rhizosphere (Supplementary Table 4). Similarly, functions central to carbon cycling from complex polymers were markedly lower in the F_Ip group, as evidenced by reduced potential for cellulolysis (log2FC = − 2.95) and xylanolysis (log2FC = − 2.96). Other affected processes included the degradation of aromatic compounds (log2FC = − 2.99) and hydrocarbons (log2FC = − 2.86), as well as the dark oxidation of sulfur compounds (log2FC = − 2.91) (Supplementary Table 4). Even the predicted relative abundance of functions related to plant pathogenesis was significantly reduced (log2FC = − 2.87). Overall, these findings point to a consistent reduction in the predicted capacity for key nutrient-cycling and organic-matter-degradation functions in the rhizosphere of *I. parviflora.*

## Discussion

This study provides taxonomic metagenomic evidence that the invasion of *I. parviflora* is associated with significant shifts in the diversity, composition, and predicted functional potential of rhizosphere bacterial communities in a temperate forest. Although the dominant bacterial phyla remained characteristic of forest soils, invaded rhizospheres exhibited higher bacterial richness, altered community composition, and reduced compositional variability compared with control soils. In addition, differential abundance and functional inference analyses indicated a widespread decline of dominant bacterial groups and a reduced predicted potential for several microbial processes. Collectively, these findings suggest that *I. parviflora* may influence belowground microbial assemblages primarily through the suppression of resident bacterial communities rather than the selective enrichment of specific microbial partners. However, because functional profiles were inferred from taxonomic composition and soil physicochemical variables were not comprehensively characterised, the mechanisms underlying these patterns remain uncertain.

### Stability of the Core Soil Microbiome

The dominance of Proteobacteria, Actinobacteria, and Acidobacteria across all samples is consistent with microbial assemblages commonly reported from temperate forest soils [15]. These phyla are globally distributed and form the structural backbone of soil microbial communities [56, 57]. At the order level, the ten most abundant taxa—including Hyphomicrobiales, Kitasatosporales, Mycobacteriales, and Burkholderiales—were present in both invaded and control plots. This persistence aligns with the concept of a resilient core microbiome that withstands moderate environmental perturbations [15, 58]. Notably, a recent study of endemic *Impatiens* species also reported rhizosphere microbiomes dominated by similar taxonomic orders [59], suggesting that a phylogenetically conserved microbial core may characterize this genus. In our system, the homogeneous soil pH (5.0–5.5), forest type, and canopy structure likely constrained large-scale taxonomic shifts irrespective of plant identity [60, 61].

### Bacterial Diversity Responses to Invasion

Alpha diversity analysis revealed a significant increase in bacterial richness in the *I. parviflora* rhizosphere (257.8 ± 3.18) compared to control soils (232.5 ± 1.98; *p* = 0.000771), whereas Shannon and Simpson diversity indices did not differ between invaded and control sites (Shannon: 3.019 vs. 3.012; Simpson: 0.899 vs. 0.898; *p* > 0.5). This pattern—higher richness without corresponding changes in composite diversity indices—suggests that invasion promoted the occurrence of additional bacterial taxa, while their low relative abundance prevented any substantial alteration of overall community diversity. These findings are consistent with a recent meta-analysis reporting limited effects of plant invasion on soil microbial α-diversity [62] and indicate that, despite an increase in the number of bacterial taxa, the general diversity structure of the community remained largely unchanged.

The increase in richness without accompanying shifts in diversity metrics points to an expansion of the rare biosphere within the invader’s rhizosphere. Although numerically scarce, rare taxa may serve as a reservoir of metabolic potential and contribute disproportionately to ecosystem functioning and stability [63, 64]. Because dominant bacterial populations appear to remain similar between invaded and control soils—as reflected by the stable Shannon and Simpson values—functional redundancy and the resilience of microbially mediated processes are likely maintained [65, 66]. However, whether the enlarged pool of rare bacteria contributes to functional stability or merely represents transient taxa stimulated by readily available root exudates remains unclear and requires functional analyses beyond taxonomic diversity assessments.

Several factors preclude an unequivocal attribution of these diversity patterns solely to the invasion process. Because *I. parviflora* was absent from control plots, rhizosphere samples were associated with different plant hosts, introducing a potential host-identity confound [33, 67]. In addition, invaded plots exhibited significantly higher soil moisture and nutrient availability (Table 1), both of which could independently promote bacterial richness, making it difficult to disentangle invasion effects from underlying habitat differences. Interestingly, nematode communities in *I. parviflora*-invaded beech forests showed compositional changes without corresponding differences in species richness [14], highlighting that the invader’s influence on soil biota may vary among organismal groups. Furthermore, documented effects on herbaceous plant diversity [5] could indirectly affect microbial assemblages through changes in litter inputs and microenvironmental conditions. Disentangling direct microbial recruitment, plant-mediated soil modification, and pre-existing site characteristics will require targeted experimental studies.

### Directional Shifts in Taxonomic Composition

Principal Component Analysis based on Aitchison distances revealed a separation between control and *I. parviflora* rhizosphere samples, with PC1 explaining 17.36% and PC2 accounting for an additional 7.17% of the total variance (Fig. 3C). Invaded samples formed a more compact cluster, whereas control samples exhibited greater dispersion, particularly along the second axis, suggesting that rhizosphere bacterial communities became more compositionally homogeneous in the presence of the invader. Such biotic homogenisation is well documented in aboveground communities following plant invasion; its occurrence belowground suggests that *I. parviflora* acts as a selective filter, reducing the range of bacterial assemblages capable of persisting in the rhizosphere [68, 69].

Differential abundance analysis identified 32 bacterial orders with significantly altered abundances (p.adj < 0.05). Notably, no orders were significantly enriched in the invaded rhizosphere; all significant changes reflected reductions in abundance. The ten most abundant orders in the dataset—Hyphomicrobiales, Kitasatosporales, Mycobacteriales, Enterobacterales, Micrococcales, Burkholderiales, Micromonosporales, Pseudonocardiales, Streptosporangiales, and Propionibacteriales—were all significantly less abundant in soils associated with *I. parviflora*, with log₂ fold changes ranging from − 2.87 to − 3.27 (p.adj = 0.0466; Supplementary Table 3). This widespread depletion, occurring in the absence of any detectable enrichment, contrasts with the selective promotion of plant growth-promoting rhizobacteria often reported for other invasive plant species [70, 71] and instead points to a broad suppression of indigenous bacterial lineages.

Several of the most strongly affected orders are associated with ecologically important soil functions. Burkholderiales, which exhibited one of the largest reductions (log2FC = − 2.99), includes numerous taxa involved in nitrogen transformations and phosphate solubilisation [72, 73]; its marked decline is consistent with the reduced nitrogen-cycling potential discussed in the following section. Propionibacteriales, commonly linked to organic matter decomposition and nutrient turnover [74], were among the most strongly depleted taxa (log2FC = − 3.04). Hyphomicrobiales, Kitasatosporales, and Pseudonocardiales—also among the dominant groups in control soils—showed similarly pronounced reductions. The consistent decline of functionally versatile taxa across phylogenetically diverse lineages suggests that the invader restructures the microbial community in a highly directional and suppressive manner, rather than simply altering the relative abundance of a limited subset of taxa.

Although the absence of any enriched order is striking, it remains possible that some rare or poorly characterised bacterial lineages increase in abundance within the invaded rhizosphere but escape detection because of limitations in current reference databases and metagenomic classification approaches [75, 76]. Consequently, while the observed pattern points to a predominantly negative effect of *I. parviflora* on the bulk bacterial community, these findings should be interpreted with caution. Further functional analyses extending beyond taxonomic composition will be necessary to fully assess the ecological implications of the observed community shifts.

### Predicted Functional Potential – a Working Hypothesis

A striking pattern emerging from this study was the consistent reduction in predicted functional potential across all 37 metabolic processes that differed significantly between groups (Supplementary Table 4). All processes, showed higher inferred abundances in control soils, encompassing key biogeochemical pathways such as nitrogen cycling (nitrogen fixation, nitrate reduction, denitrification, nitrate respiration), polymer degradation (cellulolysis, xylanolysis), aromatic compound degradation, and dark oxidation of sulfur compounds. Even the predicted relative abundance of plant pathogenic functions was significantly lower in the invaded rhizosphere.

It is essential to emphasize that these functional predictions were obtained using the FAPROTAX database [52], which assigns putative metabolic capabilities to prokaryotic taxa based on curated literature associations rather than on direct detection of functional genes in the metagenomic data. As acknowledged in the methods, direct gene-level annotation (e.g., KEGG, eggNOG, MetaCyc) was not performed, because the sequencing approach was optimized for taxonomic profiling rather than for the read depth and assembly contiguity required for robust gene-centric analysis. Accordingly, the observed patterns represent inferred ecological potential and should not be equated with measured microbial activity [36, 77]. They are best regarded as hypotheses that require validation through independent approaches such as enzyme activity assays, metatranscriptomics, or metabolite profiling.

With these caveats in mind, the predicted reduction in nitrogen-cycling functions may initially appear counterintuitive given the importance of nitrogen for plant growth. However, it can be tentatively interpreted in light of the increased nutrient availability often associated with *I. parviflora* invasion. If the invader elevates nitrogen availability through the rapid decomposition of relatively nutrient-rich litter (lower C: N ratio than native species), microbial communities may down-regulate energetically costly nitrogen-acquisition pathways [36, 78]. Such a scenario would be broadly consistent with the fluctuating resource hypothesis, which posits that increased resource availability facilitates plant invasion [79] – a pattern observed for *I. parviflora* in temperate forest understories [80]. This interpretation finds indirect support in the significantly higher Ellenberg N values in invaded plots (Table 1) and in reports of elevated soil nitrogen and carbon content in *I. parviflora*-invaded beech forests [14]. Nevertheless, Ellenberg values are inferential proxies, and without direct measurements of soil nitrogen pools and transformation rates, the link between predicted functional depletion and actual nutrient dynamics remains speculative.

Similarly, the reduced inferred potential for cellulolysis and xylanolysis may indicate a shift away from the degradation of complex plant polymers toward the utilization of more readily available carbon substrates supplied by root exudates. Such changes would favor copiotrophic bacteria over specialized decomposers of recalcitrant organic matter [15, 81]. The concomitant depletion of predicted plant pathogenic functions is consistent with the Enemy Release Hypothesis [82], which postulates that invasive plants benefit from escaping specialized pathogens present in their native range. Additionally, *I. parviflora* produces a range of allelopathic secondary metabolites, including 2-methoxy-1,4-naphthoquinone (2-MNQ) and phenolic compounds, which are released through root exudates and decomposing litter [8, 9, 83]. These compounds may selectively suppress sensitive microbial taxa, potentially contributing to the broad predicted functional attenuation observed here, a phenomenon representing a microbial dimension of the Novel Weapon Hypothesis [84, 85]. However, the specific compounds involved, their effective concentrations in the rhizosphere, and their direct impacts on microbial metabolism remain unknown for this study system.

### Limitations

Functional profiles are inferred by FAPROTAX from taxonomic composition, not from direct gene-level annotation, so they represent predicted potential and require validation. Soil characterization was limited to pH, and while Ellenberg indicator values suggest higher moisture and nutrient availability in invaded plots (Table 1), these indirect proxies cannot substitute for quantitative chemical analyses; consequently, it is not possible to disentangle the invader’s effect from pre-existing edaphic differences.

Because *I. parviflora* is absent from control plots, rhizosphere samples inevitably originate from different plant species (invader vs. a mixed assemblage of native understory plants), introducing a host-identity confound that only a common-garden or reciprocal-transplant experiment could fully resolve. Additionally, Oxford Nanopore long-read sequencing (R10.4.1) carries higher raw error rates than short-read platforms [38, 39], and Kraken‑based classification with the Standard‑8 database misses taxa absent from reference databases, a well‑documented bias in soils [75, 86]. These constraints likely contributed to spurious assignments to extremophilic taxa and to the misclassification or non‑detection of part of the community.

## Conclusions

In summary, this study provides initial metagenomic evidence that *Impatiens parviflora* invasion is associated with substantial changes in rhizosphere bacterial communities in temperate forest soils. While a stable core microbiome dominated by Proteobacteria, Actinobacteria, and Acidobacteria was maintained, invaded soils exhibited increased bacterial richness, reduced compositional variability, and widespread depletion of dominant bacterial orders. Taxonomy-based functional inference further suggested a reduced potential for several biogeochemical processes, particularly those related to nutrient cycling. However, because these functional predictions were indirect and the study design could not fully separate invasion effects from host identity and environmental variation, the observed relationships should be interpreted with caution. Future studies combining experimental approaches with direct functional measurements will be necessary to determine the mechanisms underlying these microbial shifts and their ecological consequences.

## Supplementary Information

Below is the link to the electronic supplementary material.

Supplementary Material 1 (XLSX 19.4 KB)

Supplementary Material 2 (XLSX 9.18 KB)

Supplementary Material 3 (CSV 6.38 KB)

Supplementary Material 4 (CSV 5.93 KB)

## Data Availability

The datasets generated during and/or analysed during the current study are available in the ENA EMBL-EB database under accession number PRJEB109266.
